# Design and optimization of heat pump with infrared drying for *Glycyrrhiza uralensis* (Licorice) processing

**DOI:** 10.3389/fnut.2024.1382296

**Published:** 2024-05-21

**Authors:** Lichun Zhu, Yongkang Xie, Mengqing Li, Xuetao Zhang, Xinyu Ji, Xiaoqiang Zhang, Hongbo Zhu, Junzhe Gu, Qian Zhang, Xuhai Yang

**Affiliations:** ^1^College of Mechanical and Electrical Engineering, Shihezi University, Shihezi, China; ^2^Agricultural Products Processing Research Center, Henan Academy of Agricultural Sciences, Zhengzhou, China; ^3^Engineering Research Center for Production Mechanization of Oasis Special Economic Crop Ministry of Education, Shihezi, China; ^4^Key Laboratory of Modern Agricultural Machinery, Xinjiang Production and Construction Corps, Shihezi, China

**Keywords:** heat pump with infrared dryer, numerical simulation, structure optimization, drying quality, licorice

## Abstract

A new dryer, integrating infrared and heat pump drying technologies, was designed to enhance licorice processing standardization, aiming at improved drying efficiency and product quality. Numerical simulation using COMSOL software validated the air distribution model through prototype data comparison. To address uneven air distribution, a spoiler was strategically placed based on CFD simulation to optimize its size and position using the velocity deviation ratio and non-uniformity coefficient as indices. Post-optimization, the average velocity deviation ratio decreased from 0.5124 to 0.2565%, and the non-uniformity coefficient dropped from 0.5913 to 0.3152, achieving a more uniform flow field in the drying chamber. Testing the optimized dryer on licorice demonstrated significant improvements in flow field uniformity, reducing licorice drying time by 23.8%. Additionally, optimized drying enhanced licorice color (higher L* value) and increased retention rates of total phenol, total flavone, and vitamin C. This research holds substantial importance for advancing licorice primary processing, fostering efficiency, and improving product quality.

## 1 Introduction

*Glycyrrhiza uralensis* (Licorice), is a traditional medicinal material, which has been extensively studied in the international academic field, especially in its pharmacology and biochemistry. In China, licorice also is a important Chinese herbal medicine (1), including both wild and artificially cultivated varieties. While wild licorice is predominantly found in Xinjiang, Gansu, and other regions, artificially cultivated licorice is primarily concentrated in Xinjiang, Inner Mongolia, and various parts of China (2). The key active components of licorice include flavonoids, sugars, and ascorbic acid, which have various pharmacological effects such as anti-oxidation, anti-virus, and immune regulation (3). It plays a crucial role in addressing conditions like esophageal cancer, non-alcoholic fatty liver disease, alcoholic liver injury, and inflammation, contributing positively to remission and treatment (4).

However, post-harvest, licorice is susceptible to prolonged exposure to air moisture, making it prone to various fungi that produce mycotoxins. This susceptibility significantly impacts the quality and safety of Chinese medicinal materials and their processed products (5). Currently, natural drying stands as the primary method for licorice (6), but it faces challenges due to a poor sanitary environment and susceptibility to insect and fly pollution. Moreover, the extended drying period, especially during rainy weather, often leads to widespread mold formation and a substantial loss of active ingredients in licorice. Although hot air drying (7) is widely used due to its simple mechanical structure and cost-effectiveness, it suffers from low thermal efficiency and extended heating times, resulting in a significant loss of effective components and diminished drying quality in the licorice drying process.

Among various drying technologies, infrared radiation drying is an electromagnetic radiation method with rapid heating and high heat transmission capabilities. In a study conducted by Chen and Zhang (8), different drying methods were compared for their impact on the constituents of Duchess leaves. Far-infrared drying exhibited the least impact on leaf composition, effectively preserving active ingredients. Shang et al. (9) observed that licorice samples dried with far-infrared radiation displayed a more intact internal structure, characterized by a honeycomb pattern, and exhibited higher drying quality than their naturally dried counterparts. Liu et al. (10) investigated the mechanism of infrared radiation on carrot slice dehydration and noted that it decomposes large water molecule clusters into smaller ones, reducing water viscosity and enhancing mobility, penetration ability, and migration. These characteristics contribute to higher drying efficiency when employing infrared drying.

The inverse Carnot cycle serves as a foundational framework in various heat pump drying technologies, where mechanical energy compensates for the heat extracted from a low-temperature source. For water removal, a refrigeration device is connected to a high-temperature heat source, low-temperature heat sink, and moist material. This approach offers notable advantages, including high energy efficiency and a broad, adjustable range. In a study by Chen et al. (11), fresh *Bacopa monnieri* samples were desiccated using heat pump drying. The optimal process parameters were found to be a drying temperature of 50°C, *Morinda citrifolia* material length of 4 cm, and wind speed of 1.5 m/s. Additionally, Xiong et al. (12) found that lychee fruit pomace powder subjected to heat pump drying exhibited a higher content of bonded phenols compared to its hot air-dried counterparts. Wang et al. (13) applied a heat pump dryer to enhance the structure of hawthorn cookies and developed a more efficient heat pump drying system (14), in a comparative analysis of dried mango fruits, assessed the cost-effectiveness of heat pump drying against conventional fossil-energy hot air drying. The results demonstrated that the heat pump drying method, as opposed to the traditional boiler-heated approach, reduced production energy consumption by 30.17% and carbon dioxide emissions by 32%. These findings align with China’s energy-saving and emission-reduction goals, highlighting the superiority of heat pump technology in drying and producing dried mango fruit.

In summary, the integration of infrared drying with heat pump technology represents a novel approach in dehydration drying technology (15). This method leverages the strengths of both infrared and heat pump drying to achieve efficient dehydration with substantial energy savings. Notably, this specific combination of technologies has not been previously applied to licorice drying. This research addresses the challenges of low drying efficiency and suboptimal licorice quality by developing equipment for infrared combined heat pump drying tailored for the primary processing of licorice. This technology synergistically merges heat pump drying with infrared radiation drying, aiming to conserve energy and preserve quality.

Additionally, this study delves into the relationship between drying uniformity and airflow rate distribution within the drying chamber. This aspect was numerically simulated using COMSOL software, focusing on the flow field distribution and designing a drying chamber with a more uniform flow rate to investigate the distribution law between drying layers. The goal was to refine and optimize the dryer structure, evaluating its impact on the drying characteristics and intrinsic quality of licorice. This optimization is key to enhancing primary processing techniques for licorice and establishing an effective quality preservation process during drying, a crucial step in advancing the development of this botanical resource.

## 2 General plan design

### 2.1 Complete machine structure

**Figure 1:**
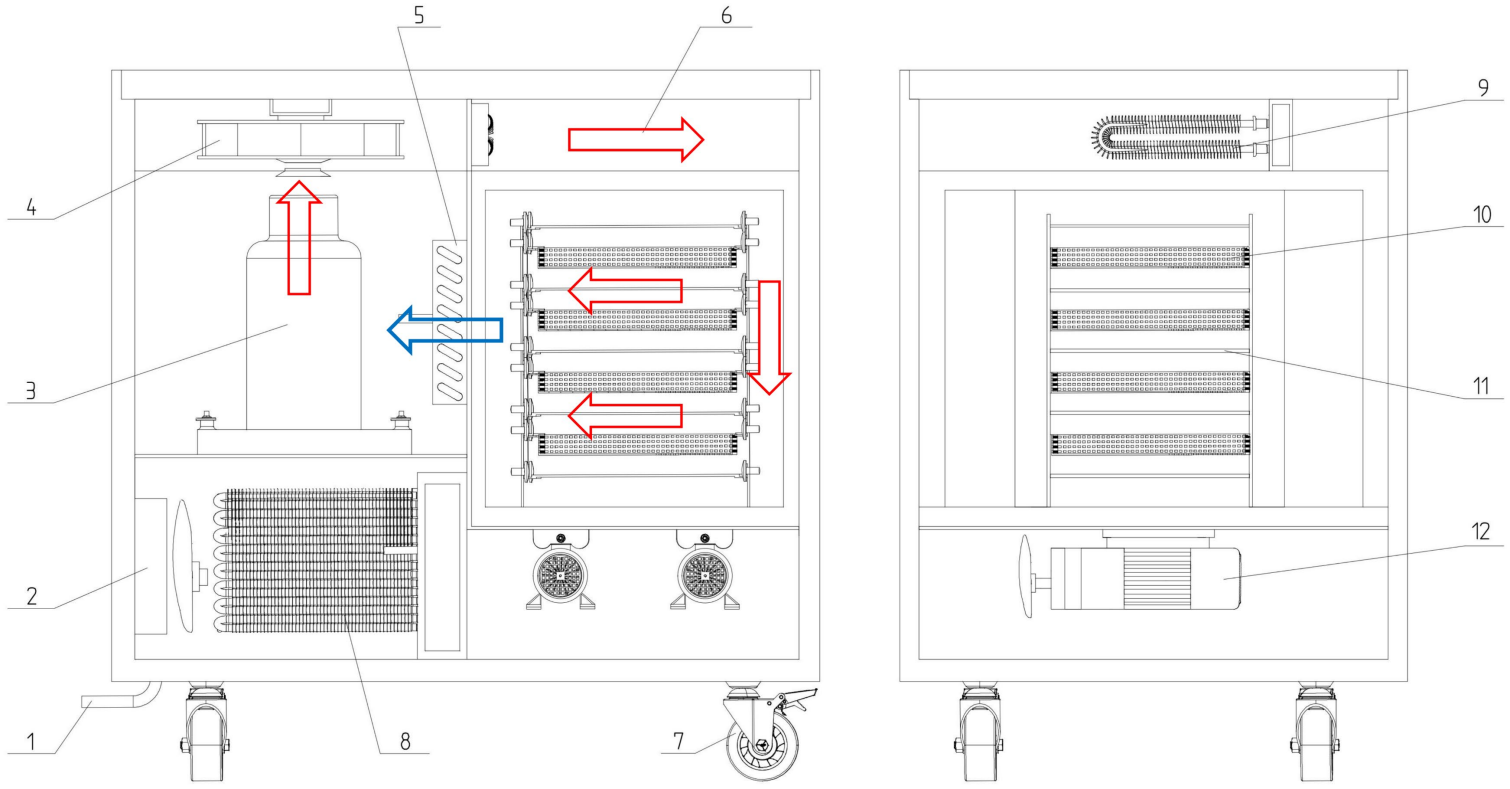
Schematic diagram of the equipment used for the infrared combined heat pump device. (1) External drain pipe, (2) external fan, (3) heat pump, (4) circulating ventilator, (5) heat exchanger 1, (6) air flow duct, (7) omni-directional wheel, (8) heat exchanger 2, (9) U-shaped heating tube, (10) material storage unit, (11) infrared heating plate, (12) dehumidifying fan.

The licorice infrared combination drying equipment with a heat pump consists of components such as the drying-box shell, heat-pump unit, moisture exhaust fan, and circulation fan, forming a three-heat-source dryer. Its heat sources include a heat pump, U-shaped heating tube, and carbon fiber infrared heating plate. The heat pump is the main heating device, and the heated air is used as the medium to heat the material. The carbon fiber infrared heating plate can dry the material by electromagnetic radiation, and its penetrating effect can accelerate the drying of the material. The U-shaped heating tube is arranged in the air duct 6, which plays the role of supplementing the heat source during the hot drying of the material. If the air temperature is not high enough or the drying needs to be accelerated, it can be opened to reheat the air flowing into the drying chamber to accelerate the drying of the material. The total structural dimensions of the casing (Figure 1) are 1,000 mm × 650 mm × 1,000 mm, and the chamber is divided into a drying chamber, an airflow duct, and a circulating air compartment by vertical and horizontal partitions. The housing’s front panel features an equipment door and a material bin door, with a human–machine-interaction touch screen positioned above the equipment door on the left side. At the rear of the equipment are the circulating air bin, heat pump unit, U-shaped heating pipes, and control unit. The entire drying chamber, corresponding to the bin door on the right side, is equipped with a material storage unit, carbon-fiber infrared heating plate, and data monitoring unit. The bottom of the box is fitted with universal wheels, facilitating easy maneuverability during loading or unloading.

### 2.2 Working principle

Upon activation, the drying apparatus initiates its heating mode through a man–machine interactive interface. This process activates the carbon fiber infrared radiation electric heating plate and the heat pump switch, allowing for the adjustment of temperature and humidity within the drying space. Preheating continues until the drying chamber reaches a stable state. Then, the licorice drying begins. The operator places a tray of licorice onto the material tray and secures the bin door. When the heat pump heats the air, the refrigerant is compressed into high temperature and high pressure gas by the compressor flows to the heat exchanger 1 (equivalent to the evaporator), and the heat exchanger 2 is equivalent to the condenser. The external fan pumps the circulating air bin where the compressor is located to the negative pressure state. The air in the drying room passes through the heat exchanger 1 into the circulating air bin and is heated. Under the pumping of the circulating ventilator, hot air is then introduced into the drying chamber via the air flow duct, heating the licorice. Concurrently, the data monitoring unit performs real-time monitoring of temperature and humidity within the chamber. The collected data are transmitted to the control unit, which processes the information and manages the operation of the heat exchange unit. The monitoring data within the drying chamber are displayed in real-time on the human-computer-interaction touch screen, enabling the operator to make informed decisions and adjust modes as necessary. The circulation fan plays a crucial role in enhancing air circulation within the enclosure and facilitating airflow through the ducts, thus improving the efficiency of the heat exchange unit. After the drying process is completed, the apparatus is powered off, and the box door is opened to retrieve the dried licorice.

### 2.3 Design and parameter determination of key components

#### 2.3.1 Infrared heating plate

**Figure 2:**
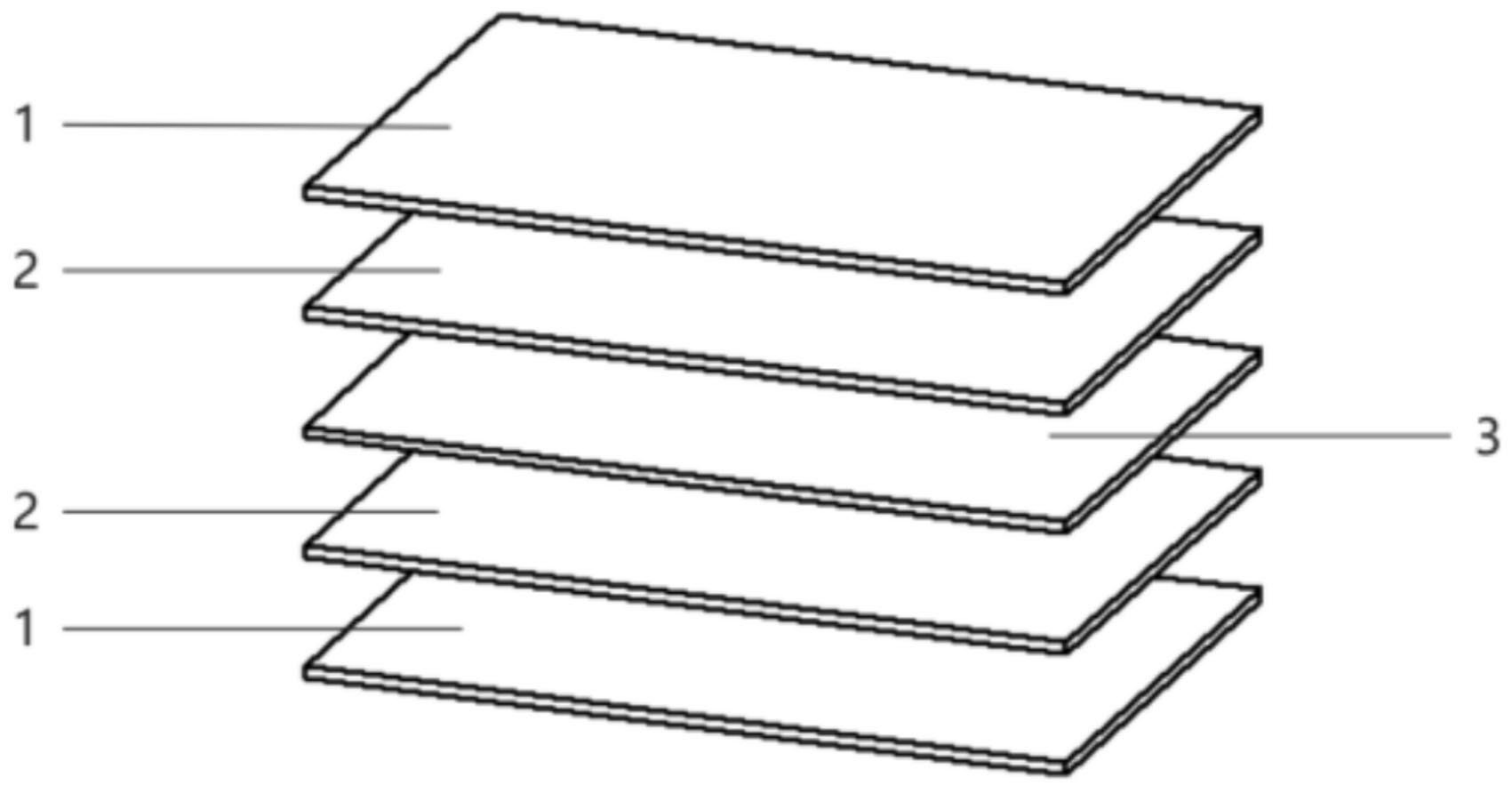
Diagram of carbon crystal infrared board. (1) Upper material layer, (2) lower material layer, (3) heating layer.

This device employs a carbon fiber infrared radiation heating panel (Figure 2), utilizing its robust heat resistance and insulating properties. The panel efficiently converts electrical energy directly into radiation energy, bypassing the need for any coating coverage. This feature ensures high heating efficiency, a simple structure, and minimal radiation spacing. The heating layer is composed of carbon fiber conductive paper. The top base layer is made of polyethylene terephthalate resin, whereas the lower base layer is made of epoxy resin glass fiber cloth, together achieving a remarkably high energy conversion efficiency. For radiation uniformity and effective heating of licorice, the device features five evenly spaced layers of infrared panels interspersed between four layers of material storage units. The power of each infrared plate is set at 0.30 kW, and the dimensions of the carbon fiber infrared radiation heating plate are 500 mm × 300 mm. This arrangement is optimized to ensure uniform heat distribution and efficient heating of the licorice.

#### 2.3.2 Heat pump dryer

The heat consumption in the licorice drying process comprises three components: the preheating heat consumption, the evaporation of internal moisture, and heat loss in the drying pipeline (16).

(1) Moisture removal during drying

The equipment processes 20 kg of licorice per batch, starting with an initial wet basis moisture content of 45% [determination by oven method at 105°C (17)], and aims for a final moisture content of 10%. The moisture removal calculation is as Equation 1:


(1) $$W_{p}=m_{1}\left(U_{1}-U_{2}\right)/\left(1-U_{2}\right)$$

where *W*_p_ represents the moisture discharged during the drying process (kg), *m*_1_ is the mass of fresh licorice before drying, 20 kg, *U*_1_ is the initial wet basis moisture content of licorice, 45%, and *U*_2_ is the final moisture content of dried licorice on a wet basis, 10%. The calculated amount of moisture to be removed (*W*_p_) is 7.78 kg.

(2) Preheating heat consumption of licorice

Considering licorice’s high water content, the specific heat capacity of water is used for calculating preheating heat. Assuming the temperature of licorice before drying is 25°C and after drying is 60°C, the preheating heat consumption (*Q*_1_) is calculated as Equation 2:


(2) $$Q_{1}=m_{1}C\left(T_{2}-T_{1}\right)$$

where *Q*_1_ represents the preheating heat consumption of licorice (kJ), *C* denotes the specific heat capacity of water, 4.2 kJ/(kg·°C) $,T_{1}$ represents the initial temperature of licorice, 25°C, and $T_{2}$ represents the final temperature of dried licorice, 60°C. The preheating heat consumption (*Q*_1_) was calculated to be 2,940 kJ.

(3) Heat consumed by evaporation of water within licorice is calculated as Equation 3:


(3) $$Q_{2}=W_{p}\lambda_{w}$$

where *Q*_2_ is the heat consumption by evaporation of internal water from licorice (kJ) and $\lambda_{w}$ is the latent heat of vaporization of water, 2,374 kJ/kg. The calculated heat consumption for evaporation (*Q*_2_) is 18,469.72 kJ.

(4) Drying process heat loss

Accounting for temperature fluctuations, heat loss occurs through pipes and the drying chamber. Assuming this loss is 20% of the total thermal energy used in drying (18), the heat loss during the drying process is calculated as Equation 4:


(4) $$Q_{3}=\left(Q_{1}+Q_{2}\right)\times 20\%$$

where $Q_{3}$ is the drying process heat loss, which was calculated to be 4281.94 kJ.


(5) $$Q=Q_{1}+Q_{2}+Q_{3}$$

Consequently, the total heat requirement (*Q*) for drying licorice amounts to 25,691.66kJ, is calculated as Equation 5.

For the purposes of energy analysis and regulation, specific equipment was selected. This includes a Beijing Haili compressor with a rated power of 2.6 kW, a WHP02830BSX-C7LU heat pump operating at 220 V, 50/60 Hz, and using refrigerant R134a.

#### 2.3.3 Drying chamber

The drying chamber is equipped with a material storage unit, carbon fiber infrared heating panels, and a data monitoring unit. Symmetrical pallets, positioned between the chamber’s two side walls, serve as bases for the material storage trays. These trays are designed with ventilation holes at the bottom to facilitate heat exchange with the material. The carbon fiber infrared heating panels are strategically placed both above and below the tray, ensuring uniform and effective heating of the material. The data monitoring unit, situated on the left side of the drying chamber wall, features temperature and humidity sensors, along with a sensor to measure the internal temperature of the material. At the chamber’s bottom, the temperature and humidity sensors are positioned between two adjacent trays, and the tip of the internal material temperature sensor extends from the side walls of the chamber to its base.

#### 2.3.4 Data monitoring and control

In the drying chamber, real-time temperature and humidity monitoring systems are installed, and the control unit contains a controller and a touchscreen. The controller is mounted on the left side of the housing, electrically linked to temperature and humidity sensors, the material’s internal temperature sensor, the exterior fan, and the circulation fan. The HMI touch panel is positioned on the front side of the enclosure, above the left equipment entrance, displaying real-time monitoring data and managing the drying stages. The touchscreen is also electrically connected to the controller.

## 3 Drying chamber flow field analysis and optimization

Computational fluid dynamics (CFD) simulation technology plays a pivotal role in rapidly determining flow field parameters, such as velocity, temperature, and pressure, within the drying chamber at various stages. CFD simulations are extensively used to enhance the structural performance of drying equipment for fruits and vegetables, offering the benefits of low simulation costs and high speeds (19). The process begins with the numerical modeling of the dryer’s interior, specifically the drying chamber. The accuracy of this model is validated against prototype data. To rectify the challenge of uneven airflow distribution observed in the prototype’s drying chamber, a multi-stage spoiler is introduced into the airflow distribution chamber. This modification, guided by CFD simulations, aims to establish a uniform flow field within the drying chamber, thereby optimizing the drying process.

### 3.1 Drying chamber air velocity field analysis

**Figure 3:**
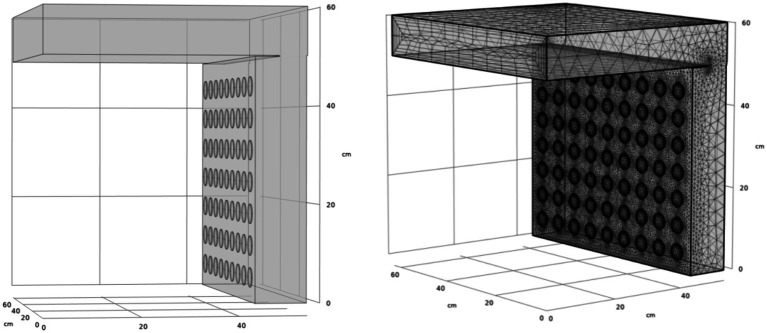
Meshing of drying chamber.

Figure 3 illustrates the numerical model of the air distribution chamber within the dryer, featuring an air inlet, equalization chamber, and air outlet. Airflow during the drying process initiates at the air inlet, progresses toward the right and downwards, and ultimately exits through the left side. The dimensions of the inlet are 630 mm × 100 mm, and the height of the equalization chamber is 600 mm. The chamber’s air outlets consist of 63 evenly spaced circular outlets, each with a radius of 20 mm and a 65 mm gap between adjacent outlets. These outlets are arranged into seven layers within the drying chamber, identified as drying layers 1 through 7, from top to bottom. Meshing for this model was performed using COMSOL Multiphysics 6.1, employing a coarser mesh to ensure convergence. The total mesh count is approximately 593,647, as depicted in Figure 3.

### 3.2 Control equations and simplifying assumptions

The flow dynamics of Newtonian fluids are primarily characterized by the Navier–Stokes equation, a fundamental principle governing the behavior of viscous, incompressible fluids is calculated as Equation 6 (20):


(6) $$\frac{\partial }{\partial_{t}}\left(\rho u_{i}\right)+\frac{\partial }{\partial x_{j}}\left(\rho u_{i}u_{j}\right)=\frac{\partial }{\partial x_{j}}\left[-p\delta_{ij}+\mu \left(\frac{\partial u_{i}}{\partial x_{j}}+\frac{\partial u_{j}}{\partial x_{i}}\right)\right]+\rho g_{i}$$

where ρ denotes the fluid density, $x_{j}$ denotes the coordinate component along the j -direction in the flow field (m), $u_{j}$ represents the mean relative velocity component along the j -direction in the flow field (m/s), δ represents the Kronecker increment, μ is the dynamic viscosity (kg/ms), and g denotes the acceleration of gravity (m/s^2^).

For turbulence calculations, the Reynolds-averaged Navier–Stokes equations (RANS) are prevalently used. In this context, a standard k-ε turbulence model, known for its robustness and adaptability to complex flow characteristics, was selected. The iterative solution utilized the SIMPLE algorithm for pressure–velocity coupling, with the relevant Equations 7–10 (21):


(7) $$\rho \left(u\cdot \nabla \right)k=\nabla \cdot \left[\left(\mu +\frac{\mu_{T}}{\sigma_{k}}\right)\nabla_{k}\right]+P_{k}-\rho \varepsilon$$

(8) $$\rho \left(u\cdot \nabla \right)\varepsilon =\nabla \cdot \left[\left(\mu +\frac{\mu_{T}}{\sigma_{\varepsilon }}\right)\nabla_{\epsilon }\right]+C_{\varepsilon 1}\frac{\varepsilon }{k}P_{k}-C_{\varepsilon 2}\rho \frac{\varepsilon^{2}}{k}$$

(9) $$\mu_{T}=\rho C_{\mu }\frac{k^{2}}{\varepsilon }$$

(10) $$P_{k}=\mu_{T}\left[\nabla_{u}:\left(\nabla_{u}+{\left(\nabla_{u}\right)}^{T}\right)\right]$$

where k represents the turbulent kinetic energy (m^2^/s^2^), μ denotes the viscosity (Pa∙s), $\mu_{T}$ denotes the turbulent kinematic viscosity (m^2^/s), $C_{\varepsilon 1}$ and $C_{\varepsilon 2}$ represent the turbulence modeling coefficients, $\sigma_{\varepsilon }$ and $\sigma_{k}$ are the Prandtl numbers for kinetic energy and kinetic energy dissipation, respectively, ε denotes the turbulent kinetic energy dissipation rate (m^2^/s^3^), and Cμ denotes an empirical constant factor.

To optimize work efficiency while maintaining accuracy and minimizing unnecessary computational efforts, several assumptions were made in this experiment:

1. The drying medium, air, was treated as an incompressible gas.
2. The inner and outer walls of the dryer were considered adiabatic, precluding any heat exchange with the external environment.
3. The influence of the material tray on airflow was disregarded.

### 3.3 Boundary conditions and evaluation indicators

The experimental setup began with defining the intake boundary conditions, assuming that the airflow direction was perpendicular to the boundary with a normal inflow velocity of 1.2 m/s. The turbulence intensity was set at a medium level (0.05), and the turbulence length scale was determined by the geometry. Pressure outlet boundary conditions were integrated with actual working conditions to estimate the outlet turbulence intensity based on the Reynolds number and turbulence intensity at approximately 5%. Due to the strong velocity gradients induced by the wall driving the flow, the flow simulation near the solid wall region was adjusted using the standard wall function approach with the no-slip wall boundary condition (22).

The velocity deviation ratio *E* was employed to assess the variance in inlet air velocity across different drying layers, aiding in analyzing the flow velocity distribution within each layer of the drying chamber. The uniformity of the velocity distribution was determined using the velocity inhomogeneity coefficient M (23), calculated as Equations 11–12:


(11) $$E=\frac{\left|\bar{V_{L}}-\bar{V_{a}}\right|}{\bar{V_{a}}}\times 100\%$$

(12) $$M=\frac{\sigma_{v}}{\bar{V_{a}}}\times 100\%=\frac{\sqrt{\frac{1}{n-1}\sum_{i=1}^{n}{\left(V_{i}-\bar{V_{a}}\right)}^{2}}}{\bar{V_{a}}}\times 100\%$$

where $\bar{V_{L}}$ represents the overall mean value of inlet air velocity for each drying layer (m/s), $\bar{V_{a}}$ represents the overall mean value of drying oven inlet velocity (m/s), $\sigma_{v}$ denotes the standard deviation, *n* denotes the point location, and $V_{i}$ denotes the velocity at each point (m/s).

### 3.4 Model validation

To validate the developed numerical model of the dryer, a comparison with actual values was necessary. Actual values were measured using a hot-wire anemometer (TES-1304, TES Electronics Industry Co., Ltd., Taiwan, China). Analog values were determined using the point probe tool in COMSOL Multiphysics. The drying test was conducted at 60°C. Before measurements, the machine underwent a 30-min warm-up phase, and wind speed measurements were initiated upon achieving operational stability. To ensure data precision, the air velocity probe was placed vertically 20 mm from each air exit. Measurements were stabilized before recording, with three readings per outlet averaged to obtain consistent data.

**Figure 4:**
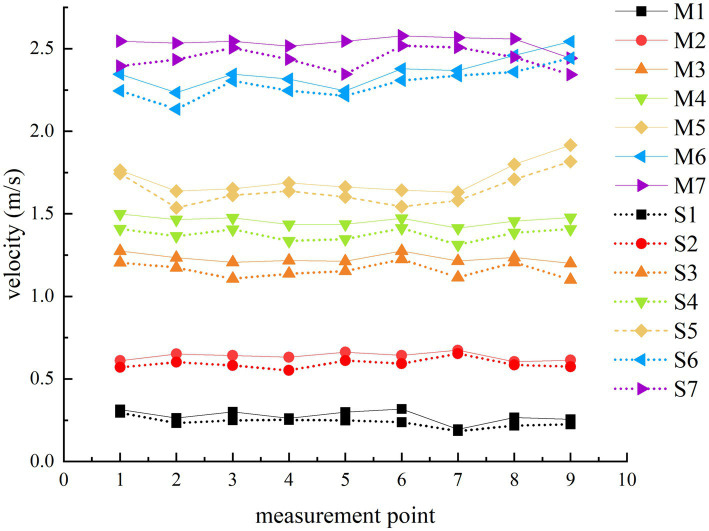
Comparison of real and simulated wind speed measurement results.

Figure 4 presents a comparison between actual and simulated wind speed measurements, revealing that the modeled values are generally higher than the measured ones. This difference is likely due to structural and sealing variations within the actual drying box, which lead to wind energy losses not accounted for in the idealized simulation scenario. Despite this, the overall trends of the simulated and measured speeds are similar. Starting from layer 1, the wind speed progressively increases, with the maximum deviation between real and simulated wind speeds in the same drying layer being 9.28%. This deviation is within the acceptable confidence level of 10% (24), affirming the numerical model’s validity for guiding future flow field optimization.

### 3.5 Flow field analysis and optimization

During the initial model validation, it was observed that the velocity in each drying layer increased progressively, contrary to the desired uniform drying conditions. Consequently, optimizing the airflow distribution chamber’s velocity flow field is essential for achieving uniform drying.

**Figure 5:**
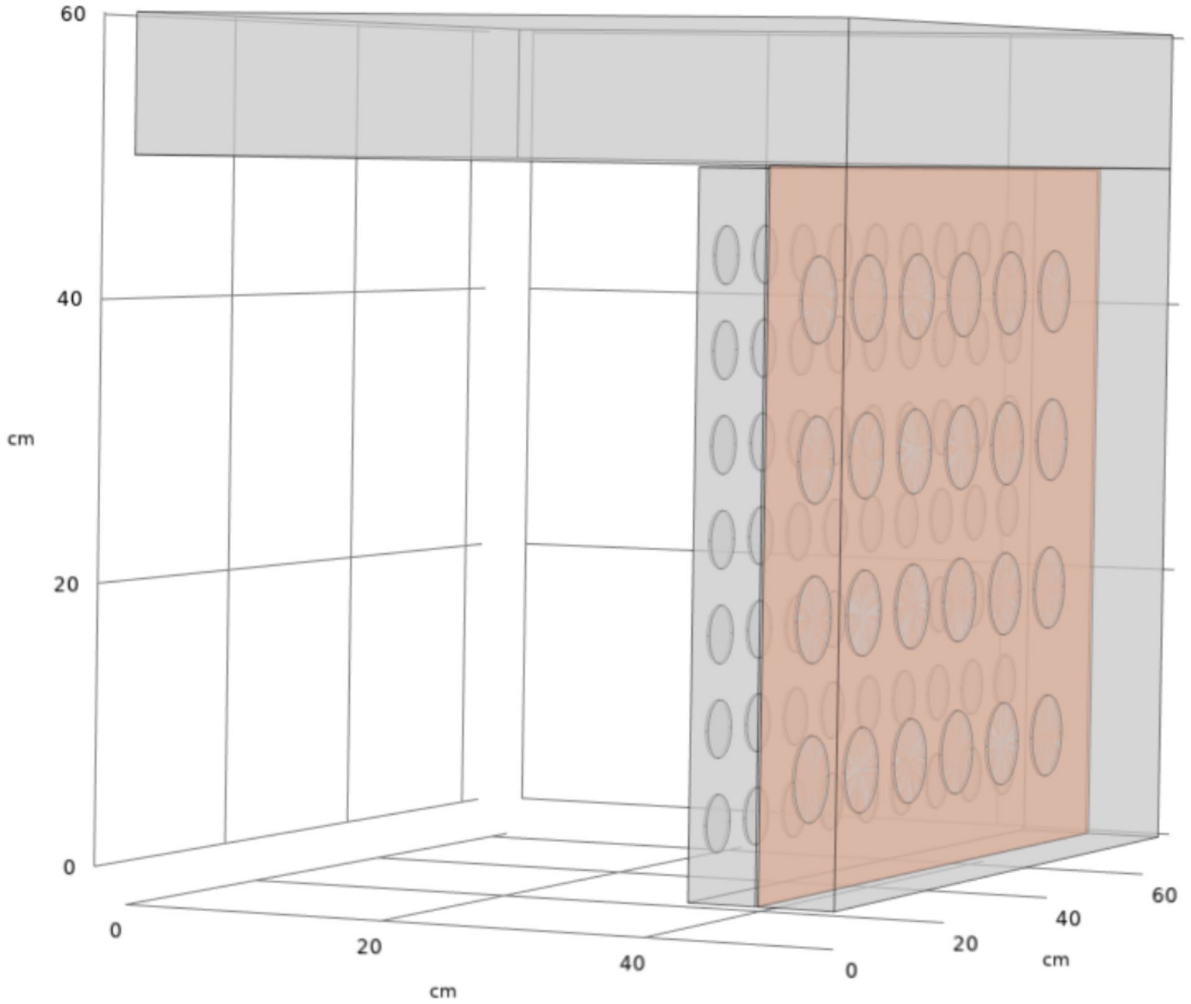
Schematic diagram of a drying chamber for adding a spoiler.

**Figure 6:**
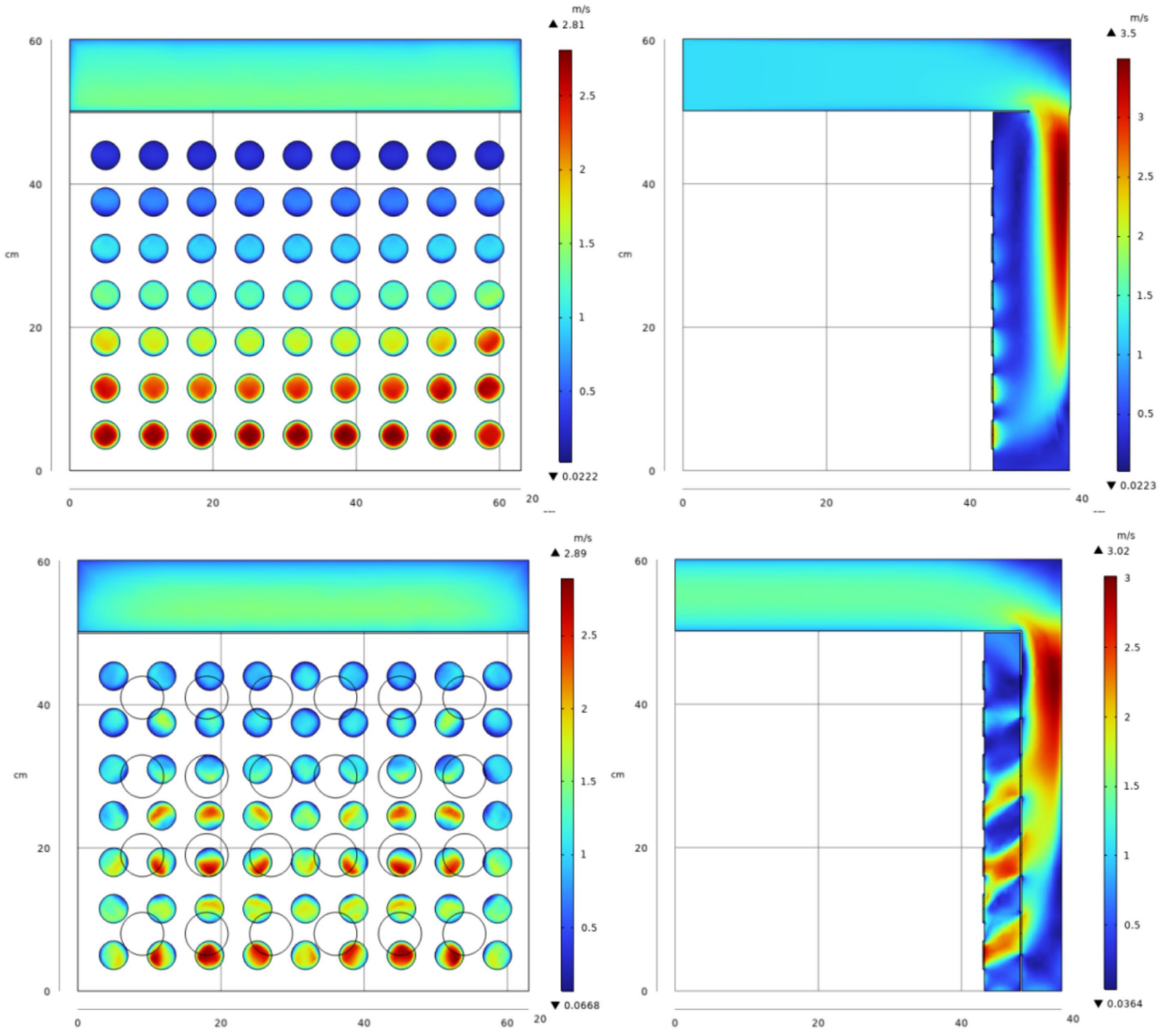
Flow field distribution before and after optimization.

Geng et al. (25) suggested that introducing a semi-cylindrical spoiler on the left side of the airflow distribution chamber could improve the uniformity of incoming air velocity across each drying layer. To explore the most effective optimization technique, a preliminary feasibility test was conducted. This involved introducing a baffle plate with four rows of 24 circular baffle holes, each with a radius of 30 mm, positioned 40 mm to the right of the air outlet (as shown in Figure 5). The average velocity of the air inlet for each drying layer was determined using the boundary probe function in COMSOL Multiphysics. Figure 6 display simulated velocity sections and graphical comparisons before and after introducing the screed plate.

The simulation comparison diagram reveals that, prior to installing the even wind plate, a portion of the drying gas enters the air distribution chamber, collides with the inner wall, flows downward, and accumulates at the chamber’s bottom, entering the lower drying layers. This results in excessively high airflow velocity in the lower drying layers, while the top drying layer receives minimal airflow, necessitating modifications to achieve the desired uniform drying conditions.

With the introduction of the air deflector in the drying system, the drying airflow is redirected downward through the leveling perforations, effectively channeling a portion of the airflow into the upper drying layer. This design alteration disrupts the vertical downward airflow, thus preventing it from converging at the bottom of the chamber. After the installation of the spoiler, calculations based on probe data indicate a significant improvement in airflow distribution. The average velocity deviation ratio for each drying layer diminishes from 0.5124 to 0.2565%, and the velocity inhomogeneity coefficient is reduced from 0.5913 to 0.3152.

Despite the uniform air plate’s contribution to flow field homogenization, the airflow in the higher drying layers remains considerably lower than that in the lower layers. Consequently, further optimization of the flow field was pursued by adjusting the dimensions of the screed. The adopted strategy involved maintaining the first row of screed holes unchanged while progressively reducing the size of the subsequent rows, as detailed in Table 1.

**Table 1:** Specific optimization scheme.

Type	Optimization program
Unoptimized	None
Feasibility study	Addition of 4 rows of 24 circular air distribution holes with radii of *r* = 3 cm.
Optimization 1	The first row of leveling holes remains unchanged, and the radii r of the remaining rows of leveling holes are 2.75, 2.5, and 2.25 cm in order from top to bottom.
Optimization 2	The first row of leveling holes remains unchanged, and the radii r of the remaining rows of leveling holes are 2.5, 2.25, and 2 cm from top to bottom.
Optimization 3	The first row of leveling holes remains unchanged, and the radii r of the remaining rows of leveling holes are 2.25, 2, and 1.75 cm from top to bottom.
Optimization 4	The first row of leveling holes remains unchanged, and the radii r of the remaining rows of leveling holes are 2, 1.75, and 1.5 cm from top to bottom.

**Figure 7:**
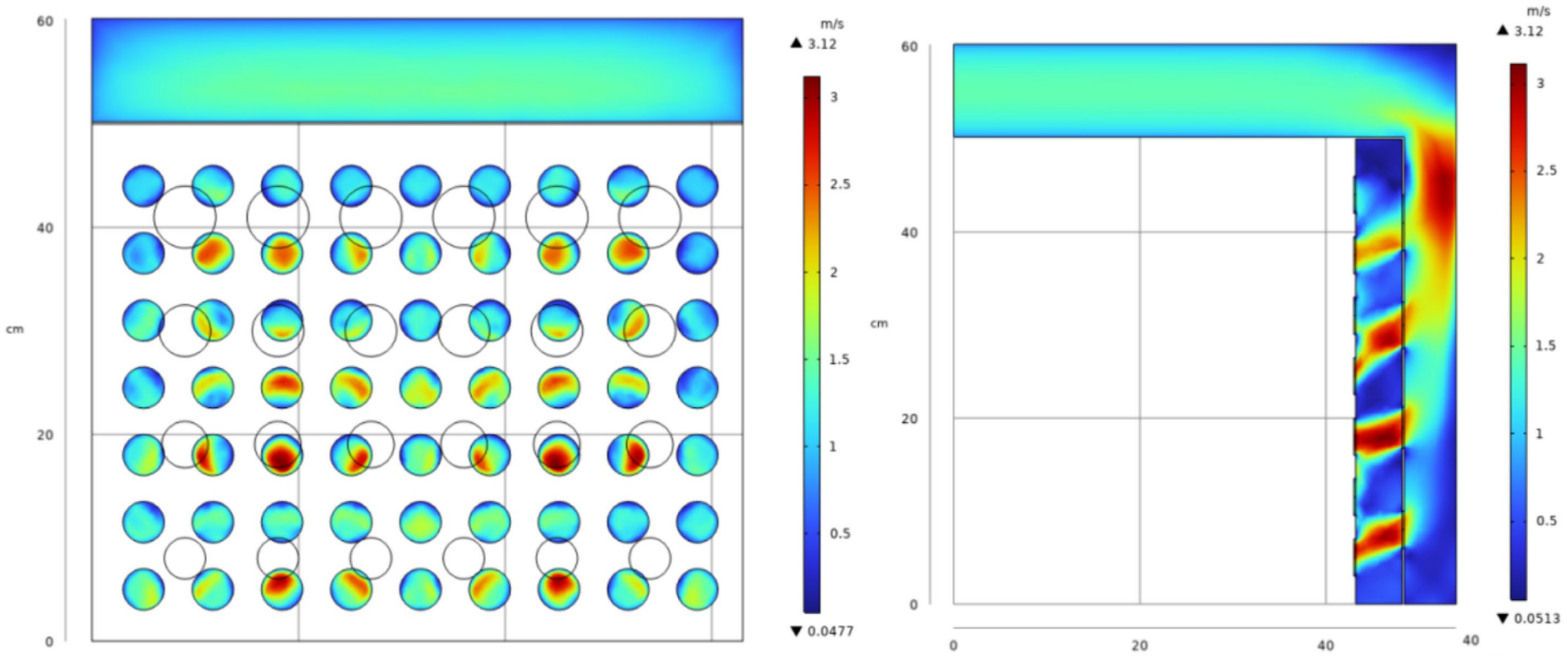
Finally optimized flow field distribution diagram.

Table 2 compiles the data from before the drying system’s optimization, the feasibility tests, and after implementing various optimization schemes. The data indicate that Optimization 2 yields the most favorable outcomes. Calculations based on the probe data show that the optimal average velocity deviation ratio is reduced from 0.5124 to 0.2565%, and the velocity inhomogeneity coefficient decreases from 0.5913 to 0.3152, which aligns with the drying uniformity criterion and greatly improves the issue of uneven airflow distribution. Figure 7 illustrates the improved velocity distribution, where the reduction in baffle hole sizes in the lower layer restricts air inflow to the lower drying layer. This stepwise adjustment in the air distribution structure successfully ensures a more balanced airflow across each drying layer, effectively resolving the issue of uneven airflow distribution.

**Table 2:** Summary of before and after optimization data.

Type	Unoptimized	Feasibility study	Optimization 1	Optimization 2	Optimization 3	Optimization 4
Average wind speed at first level (m/s)	0.2221	0.7586	0.8227	0.9454	1.0929	1.2314
Average wind speed at second level (m/s)	0.5247	0.8882	1.1855	1.402	1.6497	1.9243
Average wind speed at third level (m/s)	0.8083	0.9801	1.1479	1.1537	1.1449	1.1666
Average wind speed in layer 4 (m/s)	1.1437	1.3386	1.4612	1.4186	1.4289	1.4046
Average wind speed in layer 5 (m/s)	1.6106	1.5326	1.4612	1.5514	1.56	1.5164
Average wind speed in layer 6 (m/s)	2.0194	1.3476	1.2559	1.1503	1.0683	1.0085
Average wind speed in layer 7 (m/s)	2.1059	1.7581	1.5406	1.4947	1.4211	1.3356
Mean overall wind speed (m/s)	1.1739	1.1983	1.2562	1.2793	1.3204	1.356
First layer velocity deviation ratio E_1_	0.8107	0.3669	0.3450	0.2609	0.1722	0.0918
Second layer velocity deviation ratio E_2_	0.5529	0.2587	0.0562	0.0959	0.2493	0.4191
Third layer velocity deviation ratio E_3_	0.3113	0.18207	0.0862	0.0981	0.1329	0.1396
Forth layer velocity deviation ratio E_4_	0.0257	0.1170	0.1631	0.1088	0.0821	0.0358
Fifth layer velocity deviation ratio E_5_	0.3720	0.2789	0.1631	0.2126	0.1814	0.1182
Sixth layer velocity deviation ratio E_6_	0.7202	0.1245	0.0002	0.1008	0.1909	0.2562
Seventh layer velocity deviation ratio E_7_	0.7939	0.4671	0.2263	0.1683	0.0762	0.0150
Mean velocity deviation ratio E_A_	0.5124	0.2565	0.1486	0.1494	0.1551	0.1537
Velocity inhomogeneity coefficient M	0.5913	0.3152	0.2642	0.2490	0.2677	0.2997

## 4 Experimental verification and analysis

### 4.1 Test materials

All the samples of fresh licorice were collected from the Urals planted artificially in Qiemo County, Bazhou, southern Tianshan Mountains, Xinjiang, China. The raw materials of three-year-old licorice were extracted from the underground root section at 40 yuan/kg and stored in a refrigerator at 4°C (9). Before the test, the fine roots of licorice were removed, and the mud and sand carried in the process of harvesting were cleaned. To ensure the reliability of the results and reduce errors, licorice root strips with a similar diameter of 10 ± 2 mm were selected and sliced before drying, with a slice thickness of 4 ± 0.5 mm.

### 4.2 Instruments and equipment

In this experiment, licorice was dried using the infrared combined heat pump drying device before and after optimization. Before each test, the drying equipment needed to be preheated. This involved running the drying equipment for 30 min in advance under the set parameter conditions to achieve a stable working state, after which licorice was introduced to initiate the drying operation.

Equipment used in the experiment included a 5427R high-speed refrigerated centrifuge (Eppendorf Company), an L6-180 ultrasonic cleaning machine (Shanghai Haozhuang Instrument Co., Ltd.), a rotary evaporator (Heidolph), an Ms1602Ts 100,000th electronic analytical balance (METTLER Toledo Instruments Ltd.), and an FW type high-speed universal crusher (Beijing Yongguang Medical Instrument Factory). Other equipment included a weighing electronic balance (accuracy 0.01, JA1003 type; Shanghai Precision Scientific Instrument Co., Ltd.) and a color difference meter (Model CR-400; Japan Minolta).

### 4.3 Experimental procedure

The drying process involved selecting fresh licorice with uniform thickness, complete root shape, full hand feel, no damage, and no rot. After cleaning, the sorted licorice was cross-cut into licorice tablets with a thickness of 4 ± 0.5 mm. The tablets were then weighed using an electronic balance with an accuracy of 0.01 g. The mass of licorice tablets taken from each plate was 50 g, and they were placed on the material tray.

The specific steps of the drying process are as follows:

1. Set the relevant parameters of the test (drying temperature of 60°C, wind speed of 2.2 m/s, IR power of 675 W), press the start button, and preheat the drying chamber until the drying chamber reaches the set temperature and all indicators are stable.
2. Use an electronic balance to weigh licorice slices with a mass of about 50 g. Lay them on the tray of a drying oven that has been working steadily to allow them to dry.
3. Select the dry material layer as the test factor.
4. Every 20 min after the start of the test, weigh the sample with an electronic balance and record the measurement (each measurement process takes no more than 30 s).
5. According to the measured quality of the material (the specified quality standard is 50 g), stop the drying when the moisture content of the material is reduced to the pre-determined safe moisture content (less than 10%) (26). After taking out the material and cooling it to room temperature, store the material in a storage bag and number it (repeat each group of tests three times).
6. After all tests are completed, shut down the machine and arrange the test bench.

### 4.4 Analysis of drying characteristics

#### 4.4.1 Initial moisture content

The initial moisture content of licorice was measured by direct drying in a 105°C oven (17). The test licorice was cut into slices, then 100-g samples were weighed and placed in a 105°C oven for heating and drying. The initial moisture content is calculated as Equation 13:


(13) $$W0=\frac{M0-M\mathrm{ad}}{M0}$$

where *W*_0_ is initial moisture content of licorice (%), *M*_0_ is the initial quality of the licorice slice before drying (kg), and *M*_ad_ is the licorice slice absolute dry quality (kg).

#### 4.4.2 Safe moisture content

According to the standards of the 2020 edition of Chinese Pharmacopoeia and drying test experience (26), the safe moisture content of licorice tablets is not higher than 10%.

#### 4.4.3 Drying process

The drying process of licorice is characterized based on the curves of water ratio to drying time is calculated as Equation 14; and drying rate to dry base moisture content is calculated as Equation 15 (27):


(14) $$MR=\frac{M_{t}}{M_{0}}$$

(15) $$DR=\frac{M_{t_{1}}-M_{t_{2}}}{t_{2}-t_{1}}$$

where *MR* is the water ratio; *M_t_* is the dry base moisture content at time *t*, g/g; *M*_0_ is the initial dry base moisture content, g/g; *t*_1_ *a*nd *t*_2_ are drying times, min; *M*_*t*1_ and *M*_*t*2_ are the moisture contents of the dry base at drying times *t*_1_ and *t*_2_, g/g; and *DR* is the drying rate of the material between *t*_1_ and *t*_2_ during the drying process, g/(g·h).

### 4.5 Analysis of drying quality determination

#### 4.5.1 Color

Color (28) is one of the indexes used to evaluate food quality.

The CIELAB color meter system (also known as the L*a*b* color meter system) was employed following the method of Luan et al. (29), with slight modifications. In this method, each group of dried licorice samples was randomly taken and powdered. The color difference meter (model CR-400; Japan Minolta) was then used to determine the L*, a*, and b* values of the powder color. The total color difference value, *ΔE* value, was calculated for each sample, with each sample undergoing three parallel data measurements. The average value represented the final value, and the fresh sample served as a control. A smaller *ΔE* value indicated that the sample color was closer to the color of the initial sample, indicating better color quality.

#### 4.5.2 Browning degree

The determination of the browning degree was based on the method of Tan et al. (30), with slight modifications. This involved taking 1 g of dry licorice sample, adding 2 mL of distilled water, homogenizing it five times, centrifuging at 10,000 r/min for 30 min, and then measuring the absorbance at 420 nm using a Shimadzu UV-1900i ultraviolet spectrophotometer.

#### 4.5.3 Total phenol and total flavonoid

The total phenol content was determined using the Folin–Ciocalteu (FC) method (31), with gallic acid as the standard substance. The absorbance value of the standard solution was measured at 760 nm, and the total phenol content of the sample was expressed in gallic acid equivalents (GAE) (32). Additionally, the total flavonoid content in the sample solution was determined and expressed as the rutin equivalent (RE) in mg/g (dry base).

### 4.6 Test results and discussion

#### 4.6.1 Drying kinetics

**Figure 8:**
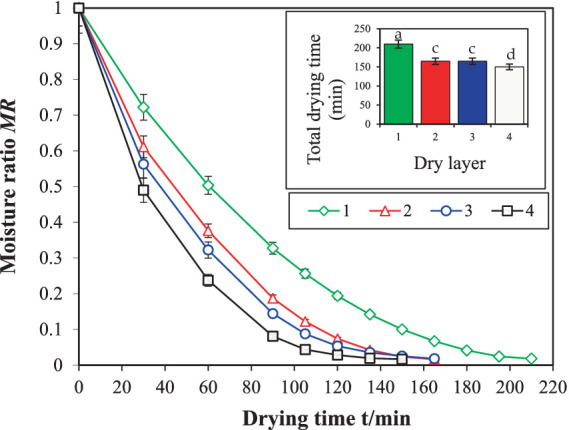
Drying kinetics curve of licorice before optimization in dryer. Different letters in the figure reveal significant differences (*p* < 0.05) according to the Duncan test.

**Figure 9:**
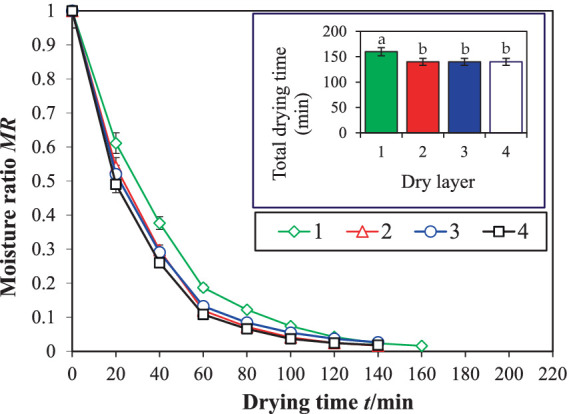
Drying kinetics curve of licorice after optimization in dryer. Different letters in the figure reveal significant differences (*p* < 0.05) according to the Duncan test.

Figures 8, 9 illustrate the influence of the drying device before and after optimization on the drying kinetics of licorice. The drying device, after installing the spoiler, had a significant impact on licorice. Post-optimization, the difference in drying time between the top and bottom layers was significantly reduced, and the drying time became almost the same as that of the middle two layers. Compared to the total drying time before device optimization, the total drying time of the entire batch of licorice was reduced by 23.8%, from 210 to 160 min. This aligns with previous research, which suggests that (33) the drying time of the entire batch of materials can be effectively shortened. The drying curves, both before and after optimization, displayed a similar trend, transitioning from steep to gentle with the change in drying time. However, the drying curves for the four drying layers after optimization were nearly identical, indicating a significant positive impact of the drying device on licorice drying uniformity between layers, consistent with previous research on licorice drying by Geng et al. (25).

#### 4.6.2 Drying quality

**Figure 10:**
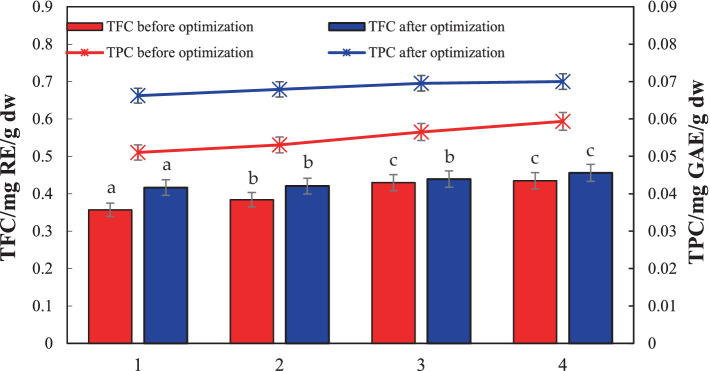
Comparison of TFC and TPC in licorice before and after optimization.

Figure 10 shows the contents of flavonoids and total phenols in licorice dried products measured before and after the optimization of the dryer. The experimental data indicates that before optimization, among the first to fourth layers of dried licorice products counted from top to bottom, the first layer has the least flavonoid and total phenol content. This could be attributed to the longer drying time of the upper layer, leading to a greater loss of heat-sensitive components, this is consistent with the study of Shi (34). The fourth layer contained the most flavonoids and total phenols. The contents of flavonoids and total phenols in the second and third layers showed little difference, exhibiting basically the same effect. This is consistent with the results of Tran et al. (35), indicating that both drying temperature and drying time have significant effects on flavonol and total phenol content.

In the licorice products dried in the optimized dryer, the contents of flavonoids and total phenols in each layer from top to bottom were essentially the same, and they were higher than those of the licorice samples dried in the corresponding layers before optimization. This demonstrates that the transformation of the drying device was effective in improving drying uniformity.

Color is a crucial quality parameter affecting consumers’ purchasing decisions, and it is an indispensable factor in evaluating dried product quality. The color parameters of fresh licorice were a = 0.15 ± 0.02, *b* = 29.18 ± 0.02, and *L* = 88.20 ± 0.07. The color comparison between dried samples of licorice before and after the optimization of the drying device is shown in Table 3. The color difference *ΔE* of licorice after optimization significantly decreased, likely due to the notably shortened drying time, which led to reduced oxidation reactions and improved color. This is consistent with the study of Li et al. (36), and the data of Browning degree also show the same changing trend.

**Table 3:** Color parameters and vitamin C retention rate of licorice before and after dryer optimization.

Parameter	Number of floors	Drying methods	
		Before	After
		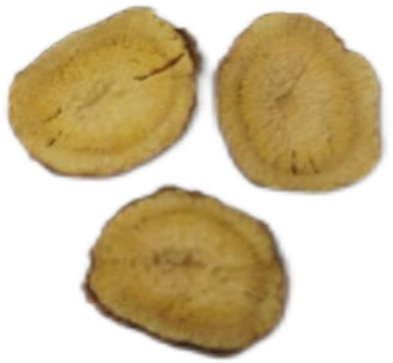	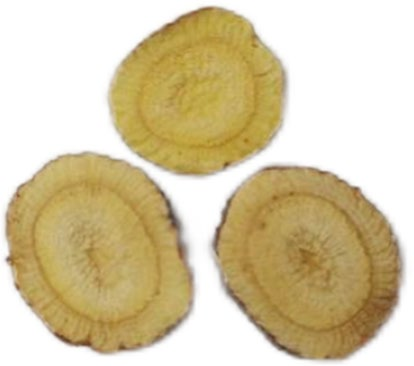
L*	1	72.41 ± 0.01^c^	73.36 ± 0.25^b^
	2	73.32 ± 0.02^b^	74.13 ± 0.15^b^
	3	74.24 ± 0.42^b^	74.89 ± 0.09^b^
	4	75.30 ± 0.02^a^	75.55 ± 0.02^a^
a*	1	1.76 ± 0.03^a^	1.56 ± 0.13^a^
	2	1.68 ± 0.02^b^	1.47 ± 0.35^a^
	3	1.35 ± 0.02^c^	1.43 ± 0.35^b^
	4	1.21 ± 0.01^d^	1.36 ± 0.02^c^
b*	1	20.92 ± 0.02^d^	23.82 ± 0.35^b^
	2	23.51 ± 0.42^c^	24.41 ± 0.29^c^
	3	23.76 ± 0.02^b^	24.82 ± 0.35^c^
	4	24.13 ± 0.01^a^	25.21 ± 0.69^a^
*ΔE*	1	17.81 ± 0.01^a^	15.78 ± 0.01^b^
	2	17.00 ± 0.01^a^	14.85 ± 0.01^c^
	3	14.95 ± 0.01^c^	14.00 ± 0.01^c^
	4	13.82 ± 0.01^d^	13.86 ± 0.11^c^
Browning index/(Abs/g d.m.)	1	1.58 ± 0.01^a^	1.15 ± 0.02^a^
	2	1.21 ± 0.01^b^	1.14 ± 0.02^c^
	3	1.17 ± 0.01^c^	1.10 ± 0.12^c^
	4	1.04 ± 0.01^d^	0.98 ± 0.20^c^
Vc retention rate	1	0.3125 + 0.25^d^	0.3571 + 0.02^c^
	2	0.3333 + 0.25^c^	0.3571 + 0.02^c^
	3	0.3571 + 0.25^b^	0.3846 + 0.02^b^
	4	0.3846 + 0.25^a^	0.4167 + 0.02^a^

Different letters in the figure reveal significant differences (*P* < 0.05) according to the Duncan test.

The experimental data also showed that the vitamin C content in licorice changed with drying time, and drying significantly reduced the vitamin C content. Table 3 displays the vitamin C retention rate in licorice compared with fresh samples. The results indicate that the vitamin C retention rate in licorice dried products after optimizing the drying device was 7.9% higher than that before optimization. This improvement may be attributed to the shorter average drying time after optimization, which avoided excessive heat loss of vitamin C. Similarly, Jiang et al. (37) found that the retention rate of vitamin C is positively correlated with drying time.

## 5 Conclusion

This study presents the development of an infrared combined heat pump dryer suitable for industrial production to reduce the adverse effects of drying licorice. COMSOL software was used to perform numerical simulations of the wind speed field in the drying chamber. The distribution law and non-uniformity coefficient of the wind speed were obtained through simulations, with the velocity deviation ratio and velocity non-uniformity coefficient serving as evaluation indexes for optimizing the size and position of the spoiler.

Upon comparison, the simulation results were found to be consistent with the test results. The accuracy and feasibility of the numerical simulation method were verified. After optimization, the average velocity deviation ratio decreased from 0.5124 to 0.2565%, and the velocity non-uniformity coefficient decreased from 0.5913 to 0.3152, resulting a more uniform flow field distribution in the drying chamber. Experimental verification with licorice showed that the optimization significantly improved the uniformity of the flow field for different air intakes and reduced the total drying time by 23.8%. The color L* of licorice after optimization was higher, and the retention rates of total phenol, total flavone, and vitamin C were also improved.

The study’s findings are significant for advancing the primary processing of licorice. However, in this study, only the velocity field was simulated and optimized, without considering the distribution and influence of the humidity field. In addition, the impact of energy consumption on the whole system can be considered in detail in the next step. Future research directions could include a detailed consideration of the impact of energy consumption on the entire system. Furthermore, future research should focus on achieving the process research with the least energy consumption and the better quality of the products.

## Data availability statement

The original contributions presented in the study are included in the article/supplementary material, further inquiries can be directed to the corresponding author.

## Author contributions

LZ: Conceptualization, Funding acquisition, Supervision, Writing – review & editing. YX: Supervision, Writing – review & editing. ML: Formal analysis, Investigation, Resources, Writing – original draft. XuZ: Investigation, Writing – original draft, Formal analysis, Resources. XJ: Investigation, Methodology, Validation, Writing – original draft. XiZ: Investigation, Writing – original draft, Methodology, Validation. HZ: Data curation, Investigation, Writing – original draft. JG: Data curation, Investigation, Writing – original draft. QZ: Validation, Writing – review & editing. XY: Validation, Writing – review & editing.
